# The Effect of Kinesio Tape Application on Clinical Parameters in Postmenopausal Women With Chronic Low Back Pain: A Randomized Controlled Study

**DOI:** 10.7759/cureus.74088

**Published:** 2024-11-20

**Authors:** Muhammet Sahin Elbasti, Kübra Akcan

**Affiliations:** 1 Physical Medicine and Rehabilitation, Elazig Medical Hospital, Elazig, TUR; 2 Medical Services and Techniques, Sirnak University, Sırnak, TUR

**Keywords:** chronic low back pain, kinesiology taping, kinesiotape, postmenopause, sleep quality

## Abstract

Background

With rising life expectancy, women are expected to spend a third of their lives in the postmenopausal stage. Consequently, focusing research on postmenopausal women is considered crucial.

Objective

This study was conducted to determine the effects of kinesiotaping (KT) and sham KT applied with exercise programs on clinical parameters such as pain, sleep, and quality of life in postmenopausal women with chronic low back pain.

Methods

A randomized controlled experimental design, characteristic of quantitative research, was utilized. The subjects consisted of 60 postmenopausal women. The visual analog scale (VAS), Roland Morris Disability Questionnaire (RMDQ), and Pittsburgh Sleep Quality Index (PSQI) were used as data collection tools.

Results

At the end of the eighth week, the mean VAS score of the experimental group was 6.7±1.0, the mean PSQI score was 5.16±1.82, and the mean RMDQ score was 8.83±2.19. In contrast, the mean VAS score of the control group was 7.2±1.5, the mean PSQI score was 6.26±1.74, and the mean RMDQ score was 10.83±3.01. At the end of the eighth week, there was no difference between the two groups in terms of VAS (p=0.128), but there was a significant difference in PSQI (p=0.020) and RMDQ (p=0.005) scales.

Conclusion

The results of this study showed that the combination of KT and exercise therapy had ameliorative effects on pain severity, dysfunction, and sleep quality in chronic low back pain experienced during the postmenopausal period.

## Introduction

Low back pain is a major health problem worldwide, causing significant physical and psychological impairment, absenteeism, and high socioeconomic costs [1]. Pain in the lumbar region lasting longer than 12 weeks is defined as chronic low back pain. In 85% of cases, nonspecific low back pain is not associated with any identifiable disease [2]. The lumbar region is the most common site of musculoskeletal pain and ranks second after headaches among the causes of pain in developed countries [3].

Among musculoskeletal disorders, low back pain is an extremely common health issue that most people, especially women, experience at some point in their lives [4]. Studies have shown that low back pain is more prevalent in women than in men, and the severity of pain increases with age [5,6]. Higher rates of low back pain in women have been linked to a decrease in gonadal hormones, such as estrogen and estradiol (reduced number of μ receptors), particularly during postmenopause, making this population more susceptible to the development of chronic pain [7]. Today, various methods, including nonsteroidal anti-inflammatory drugs, thermotherapy, electrotherapy, kinesiotaping (KT), therapeutic exercises, and manual therapy are used in the treatment of chronic low back pain [8,9]. Among these, KT is a relatively new treatment method [10]. KT creates tension that elevates the epidermis, reducing pressure on the mechanoreceptors beneath the dermis and diminishing nociceptive stimulation. KT is used in various fields, as seen in the literature [11-15]. Studies indicate that KT can improve blood and lymphatic circulation, alleviate pain, realign joints, and reduce muscle tension [16,17]. Kinesiotaping has been found effective in reducing pain and disability in patients with acute and chronic nonspecific low back pain [18-21]. Therefore, it can be utilized as a complementary method in the treatment of nonspecific low back pain.

With rising life expectancy, women are expected to spend a third of their lives in the postmenopausal stage [22]. In the literature, most of the studies on the effects of kinesiotaping include the general population while studies examining its specific effects on postmenopausal women are limited. Considering that hormonal changes in the postmenopausal period have different effects on pain perception and the musculoskeletal system, this study aims to fill this important gap by examining the clinical effects of KT and exercise combination in postmenopausal women. This study's objective was to evaluate the impact of combining KT and sham KT with an exercise program on clinical aspects like pain, sleep, and quality of life among postmenopausal women suffering from chronic low back pain.

## Materials and methods

Design of the study

This study was designed using a quantitative research method. A randomized controlled experimental design, characteristic of quantitative research, was utilized. The clinical trial registration number for this study is NCT06684145 (ClinicalTrials.gov).

Universe and sampling

**Figure 1:**
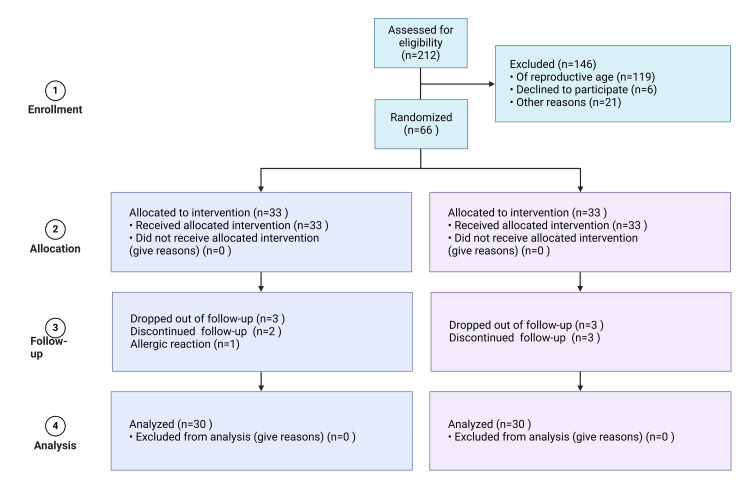
CONSORT flow diagram CONSORT: Consolidated Standards of Reporting Trials Flowchart created with www.biorender.com

The study's population comprised postmenopausal women diagnosed with chronic low back pain by a physician. The sample size and power required for the study were calculated using the G*Power Ver.3.1.9.2 program (https://www.psychologie.hhu.de/arbeitsgruppen/allgemeine-psychologie-und-arbeitspsychologie/gpower). Based on the power analysis of the comparison of visual analog scale (VAS) mean scores from Uzunkulaoğlu et al. (2018) and considering an α=0.05 risk and a 1-β=0.95 accuracy rate, the sample size needed to represent the population was determined [14]. With an effect size of 0.99 and an actual power of 0.95, it was concluded that a minimum of 27 participants were needed in each group. To account for potential sample loss, randomization included 20% more individuals per group, totaling 33 people per group and 66 overall. However, the study was completed with 60 participants, as 6 patients withdrew due to the development of allergic reactions and a desire not to continue​ (Figure 1).

The groups were randomized through simple randomization using the website www.random.org. Participants were assigned to groups according to the randomization list. They were informed that they would receive one of two different forms of KT administration but were blinded to the study hypotheses. The researcher performing the Kinesio tape application was blind to the groups because the kinesiotaping techniques and application process used in the experimental group were different from the sham taping protocol in the control group. Therefore, blinding of the Kinesio tape applicator was not possible. However, to minimize this potential bias, all assessments were performed by an independent outcome assessor who was unaware of the groups and not involved in the study. Thus, the blinding process was ensured at the assessment stage.

Postmenopausal patients with chronic nonspecific mechanical low back pain for at least three months and a VAS score of at least three were included in the study. Patients with a neurologic deficit, a history of lumbar surgery, inflammatory low back pain, spinal stenosis, spondylolisthesis, a history of cardiopulmonary disease preventing exercise, severe osteoporosis, or a skin infection in the application area were excluded.

Data collection tools

*Visual Analog Scale (VAS*)

VAS is used to subjectively measure perceived pain. It is a scale in the form of a 0-10 cm ruler, with 'no pain' at one end and 'the most severe pain' at the other, used to quantify pain intensity. The patient marks their pain level on the ruler. This marked line is then used as numerical data to determine the level of pain perception in females. Studies aimed at standardizing VAS have suggested that patients understand its vertical use more easily.

Roland Morris Disability Survey (RMDQ)

The Turkish version of the validated and reliable Roland Morris Disability Questionnaire was used to assess the patient's level of physical functionality. The questionnaire comprises 24 items, each requiring a yes/no response. 'Yes' is scored as 1 and 'no' as 0. The total score is the sum of these individual scores. On this scale, the minimum score is 0 and the maximum is 24. Higher scores indicate a greater deficiency in physical activities [23].

Pittsburgh Sleep Quality Index (PSQI)

The Turkish reliability and validity study conducted by Ağargün et al. (1996), which assesses an individual's sleep quality over the past month, includes 24 items. Of these, 19 are self-reported questions answered by the individual, and 5 are answered by the roommate or spouse [24]. The latter are not included in the scoring but are used solely for clinical information. The self-report questions cover variables related to the individual's sleep quality. The scored items comprise seven components providing insights into subjective sleep quality, sleep latency, sleep duration, habitual sleep efficiency, sleep disturbances, use of sleep medication, and daytime sleep dysfunction. Each question is rated on a scale from 0 to 3. The highest possible score on the scale is 21, with higher scores indicating poorer sleep quality.

Collection of data

Experimental Group

**Figure 2:**
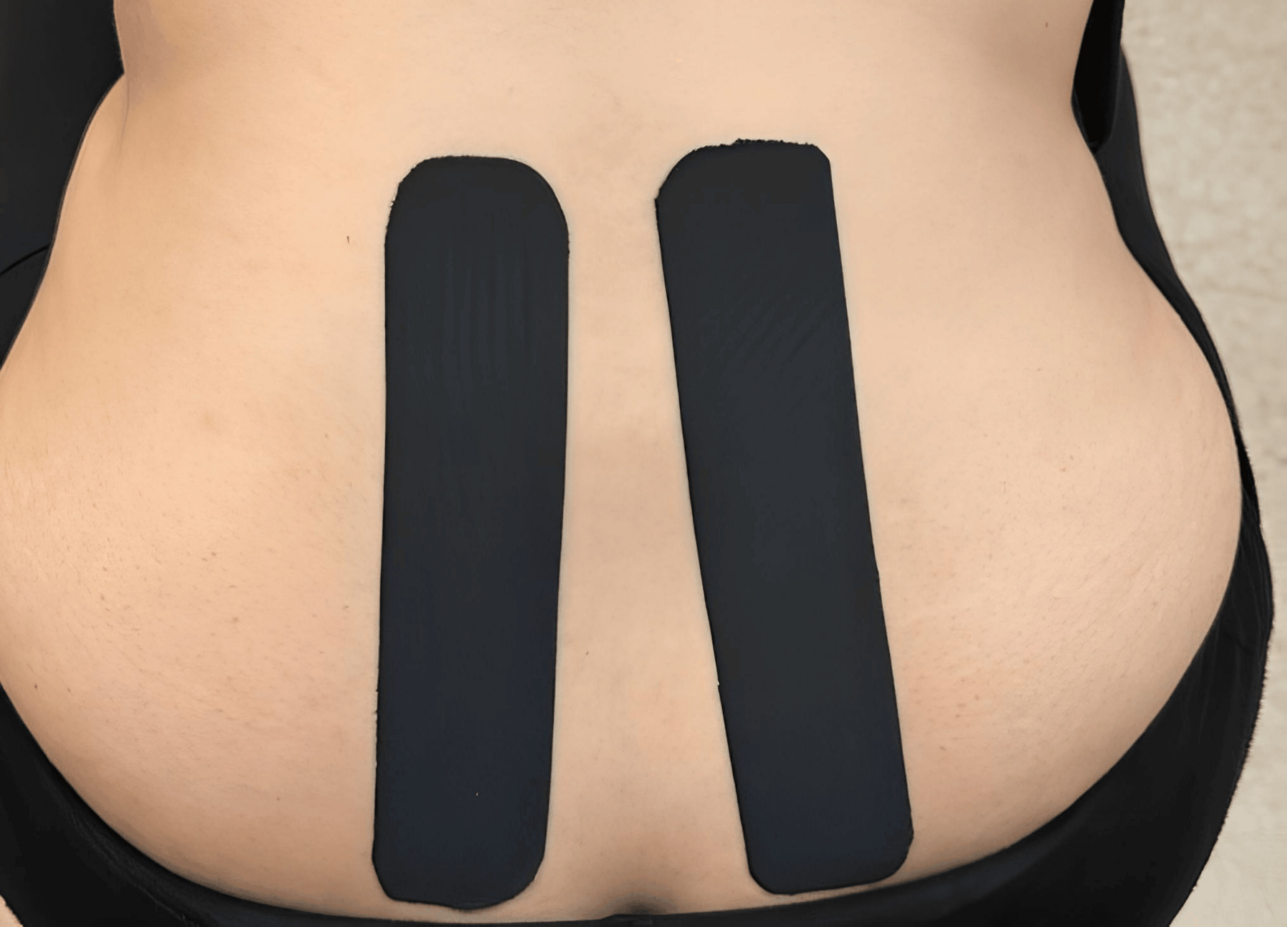
Kinesiotaping application step

Kinesio tape application was performed by a certified researcher using 5 cm x 5 cm Kinesio tape material. The standing patient, prepared in terms of clothing and skin, was asked to lean forward. While adhering the tape to the right paravertebral region, the lower end of the tape was adhered 7 cm below the sacroiliac joint at the level of the paravertebral muscles. The patient, still leaning forward, was then asked to make a slight rotation to the left; in this position, the tape was adhered upward on the paravertebral muscles without any tension. For the left paravertebral region, the procedure was reversed as compared to the right, ensuring that the tape was not stretched at all (Figure 2).

**Figure 3:**
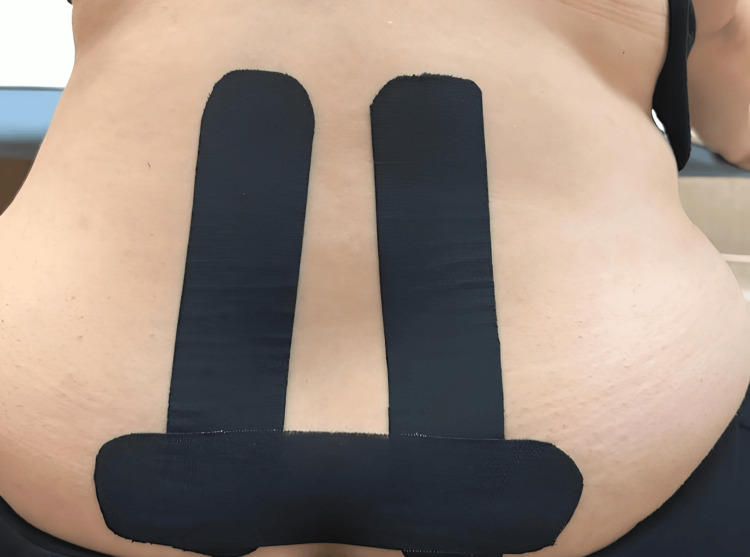
Kinesiotaping application step

The third tape was applied to the patient who was standing upright and leaning slightly forward, passing over the sacroiliac joints and parallel to the floor, with the tape stretched by 25%​​​​​ (Figure 3).

The taping treatment was applied by the same researcher 3 days a week for 4 weeks, totaling 12 tapings. Each tape remained on for 48 hours. Patients were given written schedules for their taping treatment sessions. Two measurements were taken using pre-test and post-test methods, and all evaluation methods performed before the taping treatment were repeated afterward. In addition to the kinesiotape application, patients were given a 30-day home exercise program. This consisted of passive lumbar flexion (single and double leg stretching), hamstring stretching, pelvic tilt, half sit-up, bridge, straight leg raises, and hip and back extensor strengthening exercises. Patients were instructed to perform these exercises at home three days a week for four weeks. They were also advised about avoiding movements that exacerbate pain in chronic low back pain and making appropriate behavioral changes in daily life activities (such as standing, sitting, housework, lifting weights, getting into and out of bed, etc.). Exercise schedules were provided in writing, and patients were reminded of the exercises by phone on the designated days.

Control Group

Sham Kinesio tape was applied to the patients in the control group. In the sham protocol used for the control group, Kinesio tape was adhered between the twelfth costa on both sides and the sacroiliac joint on the same side, passing over the paravertebral muscles without applying any banding technique. The third band was adhered between both sacroiliac joints without any adhesive technique. Patients were asked if they had any complaints and were informed that the tapes would be kept until the second session. The sham tape application was performed three days a week for four weeks. Two measurements were made using pre-test and post-test methods, and all evaluation methods performed before starting the taping treatment were repeated after the treatment. The final measurement was made after the last tape was removed, thus totaling 12 taping sessions. Each band was left on for 48 hours.

In addition to the sham treatment, an exercise program was implemented. Cushion exercises to strengthen the back, lumbar, gluteal, and pelvic muscles were performed for half an hour at least three days a week. A 30-day home exercise program, consisting of passive lumbar flexion (single and double leg stretching), hamstring stretching, pelvic tilt, half sit-ups, bridge, straight leg raises, and hip and back extensor strengthening exercises, was applied. Patients were instructed to perform these exercises at home three days a week for four weeks. They were also informed about avoiding movements that exacerbate pain in chronic low back pain and about making appropriate behavioral changes in daily life activities such as standing, sitting, housework, lifting weights, and getting into and out of bed. Exercise schedules were provided in writing, and patients were reminded of the exercises by phone on the designated exercise days. 

Data analysis

Descriptive statistical analyses were conducted to determine whether the data obtained during the study demonstrated a normal distribution, constituting the first step of the analysis process. In this context, it was examined whether the mean, mode, and median values of the scale scores were equal or close to each other and whether the kurtosis and skewness values fell between (-1) and (+1) [25]. Levene's test was utilized to assess the homogeneity of variances. Additionally, as the number of participants in each group was less than 50, the Shapiro-Wilk test was employed to examine the normality of the data distribution. The examination concluded that the data were normally distributed. Given the normal distribution of the data, analyses were conducted using the Unpaired Samples t-test, paired samples t-test, and analysis of variance (ANOVA) (repeated measures) tests. A p-value of less than 0.05 was considered statistically significant. ​​​​​​

The ethical dimension of research

Ethical committee approval for the study was obtained from the Health Sciences University Gazi Yaşargil Training and Research Hospital Clinical Research Ethics Committee (Decision no: 163). Institutional permission was granted by the Şırnak Provincial Directorate of Health for the study's implementation. The purpose of the study was clearly explained to the participating women, and an informed voluntary consent form was signed by each participant. The research was conducted in accordance with the ethical standards specified in the Declaration of Helsinki.

## Results

It was determined that the differences in age (p=0.132), height (p=0.949), weight (p=0.118), and BMI (p=0.211) of the participants across the study groups were not statistically significant (p>0.05) (Table 1).

**Table 1:** Comparison of participants' descriptive characteristics Group 1: Kinesiotaping+exercise, Group 2: shamtaping+exercise, BMI: body mass index p*: t-test in independent groups

	Group 1 (n =30)	Group 2 (n =30)	p*
Age (years)	56.3 ±4.3	54.73 ± 3.5	0.132
Height (cm)	160.7 ±5.5	160.80 ±6.3	0.949
Weight (kg)	69.7 ± 8.3	73.17 ±8.5	0.118
BMI (kg/cm2)	27.1 ±4.0	28.3919 ±3.7	0.211

In Table 2, the differences between the mean scores of Group 1 and Group 2 for the VAS, PSQI, and Roland Morris Disability scales at different time periods were statistically analyzed. This analysis revealed a statistically significant difference in the mean scores of the VAS, PSQI, and Roland Morris Disability scales in Group 1 before the intervention, in the fourth week after the intervention, and in the eighth week after the intervention (p<0.05). Similarly, a statistically significant difference was observed in the mean scores of the VAS, PSQI, and Roland Morris Disability scales in Group 2 before the intervention, in the fourth week after the intervention, and in the eighth week after the intervention (p<0.05). In Group 1, there was a significant improvement in the VAS, PSQI, and Roland Morris Disability Scale scores compared to the baseline at the fourth and eighth-week assessments (p<0.05). In Group 2, significant improvements were noted in the PSQI and Roland Morris Disability Scale scores at the fourth and eighth-week assessments compared to the baseline (p<0.05). For the VAS score in Group 2, a significant improvement was observed at week 4 compared to the baseline (p<0.05) while no significant improvement was noted at week 8 (p>0.05).

**Table 2:** Distribution of VAS, PSQI, and RMDQ scores of participants according to study groups and follow-up periods Group 1: kinesiotaping+exercise; Group 2: shamtaping+exercise Statistical analysis a: repeated measures ANOVA test (repeated measures); statistical analysis b: dependent groups t-test p1: 0-4. Week Dependent groups t-test. p2: 4-8. Week Dependent groups t-test p3: 0-8. Week Dependent groups t-test PSQI: Pittsburgh Sleep Quality Index; RMDQ: Roland Morris Disability Questionnaire; ANOVA: analysis of variance

	Working Groups	Day 0	Week 4	Week 8	Statistical Analysis ^a^		Statistical Analysis ^b^		
		Mean±SD	Mean±SD	Mean±SD	F	p	p ^1^	p^2 ^	p^3^
VAS	Group 1 (n=30)	8.0 ±1.5	6.2 ±1.3	6.7 ±1.0	22,463	<0.001	<0.001	0.002	<0.001
	Group 2 (n=30)	7.4±1.5	6.9±1.5	7.2±1.5	4,452	0.021	0.028	0.005	0.293
PSQI	Group 1 (n=30)	6.13±2.19	4.83±1.72	5.16±1.82	18,005	<0.001	<0.001	0.001	<0.001
	Group 2 (n=30)	6.53±1.90	6.13±1.63	6.26±1.74	6,137	0.006	0.001	0.003	0.043
RMDQ	Group 1 (n=30)	12.26±3.39	6.83±1.55	8.83±2.19	105.88	<0.001	<0.001	<0.001	<0.001
	Group 2 (n=30)	12.46±3.20	9.63±2.64	10.83±3.01	61,969	<0.001	<0.001	<0.001	<0.001

There was no statistically significant difference in the mean VAS scores on Day 0, Week 4, and Week 8 between the study groups (p>0.05). Similarly, no statistically significant difference was found in the mean PSQI scores on Day 0 between the study groups (p>0.05). However, there was a statistically significant difference in the mean PSQI scores at weeks 4 and 8 between the study groups (p<0.05). There was no statistically significant difference in the mean RMDQ scores on Day 0 between the study groups (p>0.05). A statistically significant difference was observed in the mean RMDQ scores at weeks 4 and 8 between the study groups (p<0.05) (Table 3).

**Table 3:** Comparison of pain severity and quality of life before and after treatment between groups Group 1: kinesiotaping+exercise; Group 2: shamtaping+exercise p**: t-test in independent groups VAS: visual analog scale; PSQI: Pittsburgh Sleep Quality Index; RMDQ: Roland Morris Disability Questionnaire

	Working Groups	Day 0	Week 4	Week 8
		Mean±SD	Mean±SD	Mean±SD
VAS	Group 1 (n=30)	8.0 ±1.5	6.2 ±1.3	6.7 ±1.0
	Group 2 (n=30)	7.4±1.5	6.9±1.5	7.2±1.5
	t	1,489	-1,782	-1.545
	p**	0.142	0.080	0.128
PSQI	Group 1 (n=30)	6.13±2.19	4.83±1.72	5.16±1.82
	Group 2 (n=30)	6.53±1.90	6.13±1.63	6.26±1.74
	t	-0.754	-2.998	-2.392
	p**	0.454	0.004	0.020
RMDQ	Group 1 (n=30)	12.26±3.39	6.83±1.55	8.83±2.19
	Group 2 (n=30)	12.46±3.20	9.63±2.64	10.83±3.01
	t	-0.235	-4.997	-2.934
	p**	0.815	<0.001	0.005

## Discussion

According to the findings of this study, KT is an effective supportive method in the rehabilitation of chronic low back pain. In our study, there was a significant decrease in pain intensity in the KT and exercise group at week 4 and week 8 compared to baseline. In the exercise and shamtaping group, a significant decrease in pain intensity was observed at week 4 compared to baseline but not at week 8. This result indicates that KT, when applied in addition to exercise therapy, is superior to exercise alone in reducing pain intensity for up to eight weeks. Similarly, Peñalver-Barrios et al. (2021) reported that patients who underwent a KT and exercise program showed a decrease in pain intensity at the four-week and six-month follow-ups compared to the start of treatment [9]. However, in the placebo taping and exercise group, a decrease in pain intensity was only observed at week 4. Mohamed et al. (2023) found a decrease in pain scores in the second and fourth weeks in the KT and exercise group compared to baseline [13]. Postmenopausal women experience changes in connective tissue properties, muscle function, and pain perception due to decreased estrogen levels. Kinesiotaping has been shown to alleviate pain by acting against these changes [13,15,22]. Estrogen deficiency may increase pain sensitivity. Kinesiotaping may trigger the gate control mechanism that reduces pain by stimulating mechanoreceptors [13,15,22]. Estrogen deficiency negatively affects muscle repair and recovery. Kinesiotaping may increase muscle strength in postmenopausal women by improving muscle function [26]. Hormonal changes may cause changes in posture. Kinesiotaping may alleviate discomfort by correcting posture and redistributing mechanical pressure, even for a short time [13,22]. After the vasodilatory effect of estrogen decreases, kinesiotaping may promote healing by increasing blood circulation and lymph flow [11]. The Kinesio tape Is also placed directly over the sacroiliac joints with the tape stretched by 25%, adding to the effect of the ligaments holding the joints together. This may prevent displacement of the sacroiliac (SI) joints, possibly the main source of "nonspecific" low back pain. In our study, Kinesio banding is thought to reduce pain intensity by activating blood and lymph circulation and stimulating mechanoreceptors, supporting the literature on pain control and possible mechanisms.

In this study, KT was not found to be superior to placebo taping in terms of pain relief. Similarly, Paoloni et al. (2011) concluded that there was no difference between the groups in their study that involved KT, KT+exercise, and exercise alone [26]. Furthermore, it was determined that sleep quality increased in each group at the fourth and eighth weeks compared to baseline. A comparison between the groups revealed that the sleep quality at the fourth and eighth weeks in the KT and exercise group was higher than in the sham taping and exercise group. Unlike this conclusion, Aguilar-Ferrándiz et al. (2022) reported that the sleep quality of patients with chronic low back pain who underwent KT+exercise and electrical stimulation+exercise programs increased in the fourth week compared to baseline but no significant difference was found between the groups in terms of sleep quality [27]. Ogunniran et al. (2023) reported that sleep quality in patients with chronic low back pain who underwent KT, KT+ stabilization exercise, and stabilization exercise only increased in the eighth week as compared to baseline but there was no significant difference between the study groups [28].

Finally, there was a difference in the RMDQ results at the end of treatment in both groups. A comparison between the groups showed a difference in the RMDQ results at the fourth and eighth weeks compared to the baseline. Added et al. (2016) reported a significant difference in the RMDQ results of patients with chronic low back pain who received KT + physical therapy and only physical therapy in the sixth month compared to baseline [29]. Cakmak et al. (2022) concluded that there was a significant difference in the RMDQ results in the soft tissue mobilization and KT groups compared to the pre-treatment period, but no significant difference was found between the groups [30].

Our study has some limitations. The relatively small number of participants limits the generalizability of the results to larger populations. The study evaluated outcomes only up to the eighth week, which may not capture the long-term effects of KT and exercise therapy.

## Conclusions

This study revealed that both KT and exercise therapy methods had significant ameliorative effects on dysfunction, pain intensity, and sleep quality in postmenopausal women with chronic low back pain. When KT was applied in addition to exercise therapy, a greater reduction in dysfunction and a more significant decrease in pain intensity were observed. In addition, this combination also improves sleep quality, contributing to the quality of life of patients. The findings of the study suggest that the inclusion of KT in exercise programs offers an important additional benefit in the treatment of chronic low back pain. These results suggest that the combination of KT and exercise therapy should be considered as an effective treatment modality for postmenopausal women with chronic low back pain.

## References

[1] Bachmann S, Oesch P (2013). Physiotherapy and rehabilitation for low back pain [Article in German]. Ther Umsch.

[2] de Souza Júnior JR, Lemos TV, Hamu TC (2020). Effects of Kinesio taping on peak torque and muscle activity in women with low back pain presenting fears and beliefs related to physical activity. J Bodyw Mov Ther.

[3] Oğuz H, Dursun E, Dursun N (2004). Medical Rehabilitation. Medical Rehabilitation.

[4] Hoy D, Brooks P, Blyth F, Buchbinder R (2010). The epidemiology of low back pain. Best Pract Res Clin Rheumatol.

[5] Kozinoga M, Majchrzycki M, Piotrowska S (2015). Low back pain in women before and after menopause [Article in Polish]. Prz Menopauzalny.

[6] Hoy D, Bain C, Williams G (2012). A systematic review of the global prevalence of low back pain. Arthritis Rheum.

[7] Baker JM, Al-Nakkash L, Herbst-Kralovetz MM (2017). Estrogen-gut microbiome axis: Physiological and clinical implications. Maturitas.

[8] Chou R, Deyo R, Friedly J (2017). Nonpharmacologic therapies for low back pain: a systematic review for an American College of 
Physicians clinical practice guideline. Ann Intern Med.

[9] Peñalver-Barrios ML, Lisón JF, Ballester-Salvador J, Schmitt J, Ezzedinne-Angulo A, Arguisuelas MD, Doménech J (2021). A novel (targeted) Kinesio taping application on chronic low back pain: randomized clinical trial. PLoS One.

[10] Airaksinen O, Brox JI, Cedraschi C (2006). Chapter 4. European guidelines for the management of chronic nonspecific low back pain. Eur Spine J.

[11] Doğan H, Eroğlu S, Akbayrak T (2021). Comparison of the effect of Kinesio taping and manual lymphatic drainage on breast engorgement in postpartum women: a randomized-controlled trial. Breastfeed Med.

[12] Gürşen C, İnanoğlu D, Kaya S, Akbayrak T, Baltacı G (2016). Effects of exercise and Kinesio taping on abdominal recovery in women with cesarean section: a pilot randomized controlled trial. Arch Gynecol Obstet.

[13] Pakkir Mohamed SH, Al Amer HS, Nambi G (2023). The effectiveness of Kinesio taping and conventional physical therapy in the management of chronic low back pain: a randomized clinical trial. Clin Rheumatol.

[14] Uzunkulaoğlu A, Güneş Aytekin M, Ay S, Ergin S (2018). The effectiveness of Kinesio taping on pain and clinical features in chronic non-specific low back pain: a randomized controlled clinical trial. Turk J Phys Med Rehabil.

[15] Yılmaz S, Terzioğlu F (2023). The effects of Kinesio taping and breathing exercises on pain management after gynaecological abdominal surgery: a randomized controlled study. Int J Nurs Pract.

[16] Hsu YH, Chen WY, Lin HC, Wang WT, Shih YF (2009). The effects of taping on scapular kinematics and muscle performance in baseball players with shoulder impingement syndrome. J Electromyogr Kinesiol.

[17] Kase K, Hashimoto T, Okane T (2024). Kinesio taping perfect manual: amazing taping therapy to eliminate pain and muscle disorders. Kenʼi-Kai Information.

[18] Al-Shareef AT, Omar MT, Ibrahim AH (2016). Effect of Kinesio taping on pain and functional disability in chronic nonspecific low back pain: a randomized clinical trial. Spine (Phila Pa 1976).

[19] Castro-Sánchez AM, Lara-Palomo IC, Matarán-Peñarrocha GA, Fernández-Sánchez Fernández-Sánchez, Sánchez-Labraca M, Arroyo-Morales M (2012). Kinesio taping reduces disability and pain slightly in chronic non-specific low back pain: a randomised trial. J Physiother.

[20] Kelle B, Güzel R, Sakallı H (2016). The effect of Kinesio taping application for acute non-specific low back pain: a randomized controlled clinical trial. Clin Rehabil.

[21] Macedo LB, Richards J, Borges DT, Melo SA, Brasileiro JS (2019). Kinesio taping reduces pain and improves disability in low back pain patients: a randomised controlled trial. Physiotherapy.

[22] G K P, Arounassalame B (2013). The quality of life during and after menopause among rural women. J Clin Diagn Res.

[23] Küçükdeveci AA, Tennant A, Elhan AH, Niyazoglu H (2001). Validation of the Turkish version of the roland-morris disability questionnaire for use in low back pain. Spine (Phila Pa 1976).

[24] Agargun MY, Kara H, Anlar Ö (1996). Validity and reliability of the Pittsburgh Sleep Quality Index. Turk Psikiyatri Derg.

[25] Pallant J (2020). SPSS User Guide. A Step by Step Guide to Data Analysis Using IBM SPSS. https://www.taylorfrancis.com/books/mono/10.4324/9781003117452/spss-survival-manual-julie-pallant.

[26] Paoloni M, Bernetti A, Fratocchi G (2011). Kinesio taping applied to lumbar muscles influences clinical and electromyographic characteristics in chronic low back pain patients. Eur J Phys Rehabil Med.

[27] Aguilar-Ferrándiz ME, Matarán-Peñarrocha GA, Tapia-Haro RM, Castellote-Caballero Y, Martí-García C, Castro-Sánchez AM (2022). Effects of a supervised exercise program in addition to electrical stimulation or Kinesio taping in low back pain: a randomized controlled trial. Sci Rep.

[28] Ogunniran IA, Akodu AK, Odebiyi DO (2023). Effects of kinesiology taping and core stability exercise on clinical variables in patients with non-specific chronic low back pain: a randomized controlled trial. J Bodyw Mov Ther.

[29] Added MA, Costa LO, de Freitas DG (2016). Kinesio taping does not provide additional benefits in patients with chronic low back pain who receive exercise and manual therapy: a randomized controlled trial. J Orthop Sports Phys Ther.

[30] Cakmak O, Atıcı Atıcı, E E, Gülşen M (2022). The effects of instrument-assisted soft sensation mobilization and Kinesio taping on pain, functional disability and depression in patıents with chronic low back pain: a randomized trial. Turk J Physiother Rehabil.

